# Editorial: Influence of sleep and recurrent circadian disruption on cardiometabolic health, wellbeing, and safety: from shiftwork to Monday mornings

**DOI:** 10.3389/fendo.2023.1268940

**Published:** 2023-08-22

**Authors:** Philip Cheng, Dorothee Fischer, Dayna A. Johnson, Andrew W. McHill

**Affiliations:** ^1^ Thomas Roth Sleep Disorders & Research Center, Henry Ford Health + Michigan State University Health Sciences, Detroit, MI, United States; ^2^ Department of Sleep and Human Factors Research, Institute for Aerospace Medicine, German Aerospace Center, Cologne, Germany; ^3^ Department of Epidemiology, Rollins School of Public Health, Emory University, Atlanta, GA, United States; ^4^ Sleep, Chronobiology, and Health Laboratory, School of Nursing, Oregon Health & Science University, Portland, OR, United States; ^5^ Oregon Institute of Occupational Health Sciences, Oregon Health & Science University, Portland, OR, United States

**Keywords:** circadian rhythm, circadian misalignment, jet lag, sleep disruption, sleep restriction

The modern 24-hour society is contributing to insufficient sleep and a misalignment between behaviors and internal physiology, which have negative health consequences (1). A growing literature points to increased activity during the biological night (e.g., technology use and work hours) that disrupts sleep and circadian rhythms (2, 3). Despite strong evidence for the connection between recurrent sleep and circadian disruption and poor health, exact mechanisms driving these impairments remain unclear. Therefore, this Research Topic sought to highlight research on the mechanisms by which recurrent sleep and circadian disruption impair health and wellbeing.

In this editorial, we briefly summarize the publications within the Research Topic, which span the life course and provide insight regarding sleep/circadian disruption and health.

Cardiovascular disease (CVD) is the leading cause of on-duty death among US firefighters, with work hours that are characteristically long and that extend into nighttime hours (i.e., biological night). Current evidence suggests that dimensions of sleep (e.g., sleep quality and quantity), may reduce the risk of CVD death. Romero Cabrera et al. assessed whether sleep quality and quantity were associated with cardiometabolic risk among firefighters. They found that poor quality sleep and insufficient sleep (i.e., short sleep) increased cardiometabolic risk and a higher prevalence of hypertension and obesity.

Short-sleep, however, may not be the only factor resulting in poor health outcomes, particularly given the U-shaped relationship between sleep duration and health. Li et al. sought to examine if the U-shaped relationship is found in a meta-analysis of studies in populations of middle-aged and older adults. Incidence of depression was related to sleep duration in a U-shaped dose–response manner, with both shorter and longer sleeping durations conferring increased risk of depression compared to 7 hours of sleep. The findings from both studies reinforce the importance of sleep duration as a risk factor for health and depression.

Insufficient sleep duration is a hallmark problem in night shift workers. Over 20% of the US workforce work shift work hours, including sleep and circadian disrupting shifts (4). Chronic sleep restriction and recurrent circadian disruption (e.g., rotating shift work) can increase an individual’s risk of cardiovascular (5) and kidney disease (6), though mechanisms remain unclear. McMullan et al. utilized a 32-day in-laboratory protocol in which all behaviors (e.g., eating, activity, and sleep) were equally spread across all circadian phases, thereby desynchronizing the influence of sleep and circadian phase on physiological outcomes (i.e., “forced desynchrony”). Additionally, half of the participants were randomized to a sleep restriction condition, thereby allowing the examination of sleep restriction vs circadian disruption on cardiovascular and renal outcomes. They find that sleep restriction increases blood pressure and that renal outcomes differ depending on circadian timing versus behavioral influences; these results may provide mechanistic insights into contributing factors of CVD and renal impairments observed in shift work.

In addition to increased risk of CVD, shift workers also exhibit increased risk for poor glucose tolerance (7). While prior studies indicate that sleep restriction decreases insulin sensitivity and glucose tolerance, precise mechanisms are unclear as sleep restriction is often accompanied with circadian disruption. Using a forced desynchrony protocol, Yuan et al. aimed to experimentally dissociate sleep restriction from circadian disruption and examine its contribution to glucose tolerance in healthy adults. They found that minimizing circadian disruption during sleep restriction may mitigate adverse outcomes in glucose tolerance, perhaps due to the preservation of slow wave sleep when circadian disruption was minimized. These findings further emphasize the importance of circadian rhythms to health.

Authors within this Research Topic also explored how sleep and circadian processes interact with hormone levels. Upon awakening, humans excrete a bolus of cortisol (8), referred to as the cortisol awakening response (CAR). Concurrently, cortisol also follows a robust circadian rhythm, peaking in the early morning hours (9). However, how the CAR is influenced by circadian timing is unknown. Using two forced desynchrony protocols, Bowles et al. measured the CAR as participants were awakened at differing circadian times. They report that the CAR follows a circadian rhythm, peaking in the morning and a nadir in the afternoon. These findings have implications for shift workers that awaken at differing circadian phases and for individuals that use cortisol as a diagnostic tool and thus need to consider circadian timing. Molzof et al. examined 24-hour rhythms in insulin and leptin levels among day and nightshift nurses. The authors found that nightshift nurses, compared to dayshift, had higher insulin and leptin levels across the 24-hour day. Moreover, nurses who consumed most of their calories during the nighttime on workdays exhibited higher 24-hour insulin and leptin levels compared to nurses who predominantly ate during the daytime. These findings suggest shiftwork and/or eating at night may increase risk for elevated insulin and leptin levels. Thus, eating during the daytime on workdays may reduce the negative effects of shiftwork on metabolism, but this should be further studied in a larger sample.

Last, previous studies have found that children’s body mass index (BMI) increases in the summer, but Moreno et al. sought to determine whether this increase is mediated by seasonal changes in height or weight. They found evidence of seasonality in children’s height but not weight gain; children gained height at a slightly faster rate during the school year compared to the summer, but the rate of weight gain did not differ across seasons. Comparing different BMI trajectories throughout elementary school, the authors suggest that seasonality in height may be associated with developing/maintaining a healthy weight status and that children who are at greatest risk of developing an unhealthy weight status would likely benefit from obesity prevention interventions during both the school year and summer.

The current Research Topic spans across a wide-range of professions, includes in-laboratory and fieldwork protocols, and identifies several important physiological outcomes that are all directly impacted by sleep and circadian disruption. These studies highlight how common events and behaviors, such as shift work, influence health and wellbeing. We believe that these important contributions to the field will inform intervention strategies including policy changes, behavioral interventions, and personalized medicine as the world experiences sleep and circadian disruption daily.

## Author contributions

PC: Conceptualization, Writing – original draft, Writing – review & editing. DF: Conceptualization, Writing – original draft, Writing – review & editing. DJ: Conceptualization, Writing – original draft, Writing – review & editing. AM: Conceptualization, Writing – original draft, Writing – review & editing.
